# Measuring the impact of anonymization on real-world consolidated health datasets engineered for secondary research use: Experiments in the context of MODELHealth project

**DOI:** 10.3389/fdgth.2022.841853

**Published:** 2022-09-01

**Authors:** Stavros Pitoglou, Arianna Filntisi, Athanasios Anastasiou, George K. Matsopoulos, Dimitrios Koutsouris

**Affiliations:** ^1^ Computer Solutions SA, Research & Development Dpt., Athens, Greece; ^2^ School of Electrical and Computer Engineering, National Technical University of Athens, Athens, Greece

**Keywords:** electronic health records, harmonization, anonymization, information loss, real data

## Abstract

**Introduction:**

Electronic Health Records (EHRs) are essential data structures, enabling the sharing of valuable medical care information for a diverse patient population and being reused as input to predictive models for clinical research. However, issues such as the heterogeneity of EHR data and the potential compromisation of patient privacy inhibit the secondary use of EHR data in clinical research.

**Objectives:**

This study aims to present the main elements of the MODELHealth project implementation and the evaluation method that was followed to assess the efficiency of its mechanism.

**Methods:**

The MODELHealth project was implemented as an Extract-Transform-Load system that collects data from the hospital databases, performs harmonization to the HL7 FHIR standard and anonymization using the k-anonymity method, before loading the transformed data to a central repository. The integrity of the anonymization process was validated by developing a database query tool. The information loss occurring due to the anonymization was estimated with the metrics of generalized information loss, discernibility and average equivalence class size for various values of k.

**Results:**

The average values of generalized information loss, discernibility and average equivalence class size obtained across all tested datasets and k values were 0.008473 ± 0.006216252886, 115,145,464.3 ± 79,724,196.11 and 12.1346 ± 6.76096647, correspondingly. The values of those metrics appear correlated with factors such as the k value and the dataset characteristics, as expected.

**Conclusion:**

The experimental results of the study demonstrate that it is feasible to perform effective harmonization and anonymization on EHR data while preserving essential patient information.

## Introduction

Electronic Health Record (EHR) systems are being increasingly adopted to represent various data types, such as patient medical histories, laboratory test results, medication, demographics, billing records and diagnosis codes. EHR systems are the building blocks of Health Information Exchange (HIE) networks, enabling the sharing of data and information about patients' medical and health history (1–3).

EHRs surpass many existing registries and data repositories in volume, offering a window into the medical care information of a diverse population. Their effectiveness when reused for the purpose of clinical research is proven in various instances (4–7). However, their reuse has been limited due to issues such as its high dimensionality, heterogeneity, incompleteness, noise and errors, and redundant terminology (4, 5).

Interoperability is a crucial requirement for the efficiency of healthcare information systems and the utilization of health data for clinical research. The related concept of data harmonization aims to transform heterogeneous data into a standard format using computational approaches such as lexical and semantic mapping, enabling the integrative analysis of the data and, therefore, enhancing the statistical power of the clinical studies which make use of such data. Health Level Seven (HL7) is currently the most widely used set of standards for the structure and exchange of clinical data (8).

Anonymization is another essential issue regarding the secondary use of clinical data. Patient data must be disseminated without compromising their privacy against threats such as identity, membership and attribute disclosure (2). Data privacy protection can be pursued with methods such as encryption, authentication, and de-identification, which however can be inapplicable or insufficient in preserving confidential information. For example, the removal of data identifiers such as each individual's name and social security number does not prohibit their possible reidentification through the linkage of other data attributes. To prevent such attacks, the concept of k-anonymity, as well as its extensions l-diversity and t-closeness, have been proposed (9, 10).

The k-anonymity concept, introduced by Samarati and Sweeny (11), focuses on reducing data granularity. A dataset is k-anonymous if each record is indistinguishable from at least k−1 records with respect to specific identifying attributes. A quasi-identifier (QI) set is a minimal set of dataset attributes that can be joined with external information to re-identify individual records. K-anonymity requires that each equivalence class EQ (i.e., a set of records that are indistinguishable from each other with respect to the QI set) contains at least k records. K-anonymity can be provided using suppression and generalization techniques. Suppression involves replacing a portion of the original data with a special selected value to suggest its nondisclosure, while generalization focuses on replacing the values of an attribute with less specific but consistent values. K-anonymity is considered as the “bedrock” anonymization algorithm and is used as a foundation process, even in the rare case that the overall privacy it provides could be considered inadequate, allowing the potential disclosure of sensitive attributes that lack diversity through the use of background knowledge (11–16).

Given the sensitive nature and complexity of clinical data, a systematic overall approach is needed for their secondary use, examples of which can be found in the literature. Ciampi et al. (17) proposed an architecture for the extraction, transformation and loading of clinical data, which incorporates de-identification and standardization to the HL7 CDA and FHIR formats (17). Somolinos et al. (18) proposed a pseudonymizing system developed according to the ISO/EN 13606 standard for facilitating the exchange and secondary use of data, allowing the total or partial anonymization of EHR extracts (18, 19). Quiroz et al. (20) developed an SQL-based ETL framework for the conversion of health databases to the OMOP CDM (20, 21). Ong et al. (22) developed a GUI-based ETL system for the conversion of data to the OMOP CDM (22).

**Figure 1:**
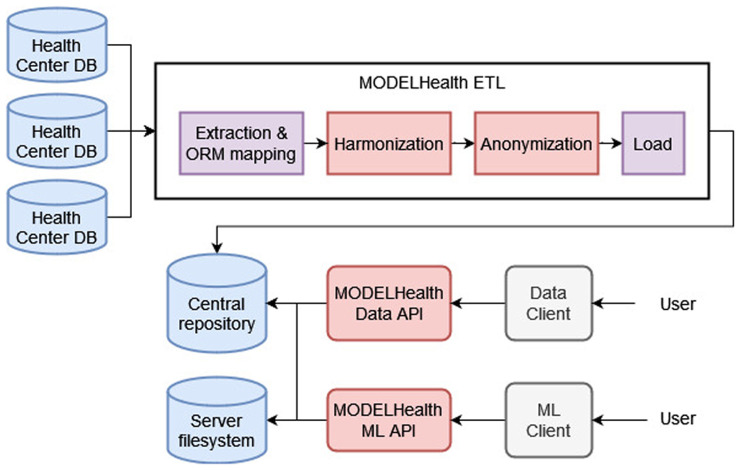
The components of the MODELHealth project.

This paper proposes an integrated solution to the problem of clinical data reuse that has been implemented in the context of the MODELHealth project. The project is based on an ETL system that extracts EHR data from several hospital databases (Section 2.1), transforms the data by performing harmonization to the HL7 FHIR standard and anonymization with the k-anonymity method (Sections 2.2, 2.2.1, 2.2.2), and loads the transformed data to a central, document-based repository (Section 2.3) (23, 24). The data used is raw EHRs from selected Greek hospital databases regarding patients, hospitalization encounters, medical procedures and observations, diagnostic reports and locations. An essential objective of the MODELHealth project has been the utilization of the transformed clinical data as input to predictive models. This goal was met by developing two public-facing REST Application Program Interfaces (Data API, Machine Learning API) and client software (Data Client, ML Client). The Data API and Client serve the purpose of making the transformed data stored in the central repository available to the interested users, while the Machine Learning API exposes the functionality of trained and validated machine learning models to the interested users. The information loss that occurred due to the anonymization was evaluated using three metrics, described in Section 2.4. The components of the MODELHealth project were developed in the Python programming language, and are depicted in Figure 1.

## Methods

### Extraction

The data extraction process involves the automated extraction of data from three hospital databases and their mapping to relational objects that reflect the database schema with the use of the SQLAlchemy Object Relational Mapper software. Implementing the MODELHealth ETL process included versioning, allowing the additive extraction, processing and loading of the data in several points in time. Each version includes all the data extracted from a health unit database until that time point. The primary key value of the last extracted record is stored for every version and every database table so that future execution of the ETL process will take into account only the new records. The detailed ER diagrams of the relational database tables from which the EHR data originated can be seen in Supplementary Figure S1.

### Transformation

#### Harmonization

The harmonization process refers to mapping the extracted data from the form of relational objects to FHIR (Fast Healthcare Interoperability Resources) ontology objects. FHIR is a RESTful API using the HTTP protocol and leveraging the HL7 Reference Information Model (RIM). FHIR defines a system of clinical, administrative, financial and infrastructure resources, its ontologies being organized in the clinical, financial, specialized, base and foundation categories (25–30).

The harmonization of the extracted data has been achieved with in-house software. First, the relational data are converted to the corresponding FHIR ontologies through custom specialized programming libraries and transformative functions related to the database schema from which the data originated. FHIR data were converted to the JSON (JavaScript Object Notation) format, as this is the preferred representation of the standard. The main FHIR entities incorporated were the Patient, Observation, DiagnosticReport, Encounter and Location ontologies. Supplementary Figure S2 depicts the FHIR entities according to which the relational data were harmonized.

#### Anonymization

The anonymization process involves modifying several fields in a given dataset to prevent the individuals' reidentification. In the scope of this project, anonymization of the harmonized EHRs was carried out using Mondrian, a greedy algorithm that implements k-anonymity through multidimensional recoding and applies to both categorical and numeric data. Mondrian performs k-anonymization of a given dataset with logarithmic worst-case time complexity in two stages. The first stage focuses on partitioning the given dataset on several multidimensional regions covering its domain space by applying a recursive algorithm similar to the ones used to construct kd-trees. The second stage focuses on applying re-coding functions to the dataset, formulated using summary statistics from each region (31).

The data fields subjected to anonymization were the birthDate and address attributes of the Patient FHIR ontology and the longitude and latitude corresponding to the address. Each address was translated to longitude and latitude coordinates through the OpenStreetMap API, which were then added as numerical fields to the patient record and were included in the anonymization process (32). Supplementary Figure S3 depicts an example of the anonymization of a sample subset of male patient records, which was subjected to the ETL process and stored in the document-based database MongoDB (see Section 2.3). A sample harmonized, non-anonymized record is depicted at the top, with the FHIR id, maritalStatus fields, as well as the _id field, which serves as a primary key for MongoDB, having been suppressed for clarity. A sample anonymized record using k = 5 is displayed at the bottom, having used the FHIR fields “address”, “birthDate”, as well as the added fields “ord_latitude” and “ord_longitute” as QI attributes.

### Loading

The loading process involved the transmission of the transformed data through a streaming process and their subsequent storage to the central repository. Data was streamed in predefined-sized packages through a TCP/IP connection. The central repository was implemented with the non-relational database MongoDB, in which every record is stored in the BSON format. MongoDB is a fitting choice for storing and retrieving JSON documents, as it is designed to handle effectively document-oriented, semi-structured data (33).

### Information loss evaluation

The impact of the anonymization on the harmonized EHR data was estimated using the metrics of generalized information loss, discernibility and average equivalence class size.

Generalized information loss (GIL) captures the penalty incurred when generalizing a specific attribute by quantifying the fraction of the generalized domain values. GIL for an anonymized table T* was calculated according to Equation (1), where T is the original table, i = 1,…,n corresponds to an attribute, j = 1,…,|T| corresponds to a table record, U_i_, L_i_ are the upper and lower values of each arithmetic attribute i, U_ij_, L_ij_ are the upper and lower values of arithmetic attribute i for the equivalence class the record j belongs in, Ni is the number of different values for each categorical attribute i and N_ij_ is the number of different values for categorical attribute i in the equivalence class the record j belongs in (34–36).

The discernibility metric (DM) measures how indistinguishable a record is from others by assigning a penalty to each record, equal to the size of the equivalence class in which it belongs. DM for an anonymized table T* was calculated according to Equation (2), where |EQ| is the number of records of the equivalence class EQ (31, 36, 37).

The average equivalence class size (C_AVG_) measures how well the created equivalence classes approach the best case, where each record is generalized in an equivalence class of k records. It was calculated according to Equation (3), where |T| is the number of table records, |EQs| is the total number of equivalence classes created in the anonymized table T*, and k is the minimum equivalence class size allowed (31, 37).


(1) $$\begin{array}{c} \mathrm{GIL}(T*)=\frac{1}{|T|n}\times \sum_{i=1}^{n}\sum_{j=1}^{|T|}\\ \{c\frac{U_{\mathrm{ij}}-L_{\mathrm{ij}}}{U_{i}-L_{i}},\;\mathrm{if}\;i\;\mathrm{is}\;\mathrm{arithmetic},\frac{N_{\mathrm{ij}}-1}{N_{i}-1},\;\mathrm{if}\;i\;\mathrm{is}\;\mathrm{categorical}\; \end{array}$$

(2) $$\;DM\left(\;T*\right)=\underset{\forall \;EQs.\;t.\left|\;EQ\right|\geq \;k}{\sum }{\left|\;EQ\right|}^{2}$$

(3) $${\;C}_{\;AVG}\left(\;T*\right)=\frac{\left|\;T\right|}{\left|\;EQs\right|\;k}$$

The information loss evaluation has been applied to experimental datasets originating from three hospital databases. More specifically, the patient data populating the table CARE_PERSON of three hospital databases were subjected to the ETL process for the k values 5, 10, 15, 20. The transformed datasets S_1_, S_2_, S_3_ correspond to the three origin database schemas, while the dataset S_123_ constitutes the union of S_1_, S_2_, S_3_. The four datasets were evaluated in terms of the information loss that occurred during the anonymization stage using Equations (1–3). The technical characteristics of the datasets S_1_, S_2_, S_3_, S_123_ are presented in Table 1.

**Table 1:** The number of records (|T|) and the size in GBs of the tested datasets S_1_, S_2_, S_3_, S_123_ for all tested k values.

Dataset\k	|T| after ETL				Dataset Size (GB) after ETL			
	5	10	15	20	5	10	15	20
S_1_	54,003				0.009	0.012	0.014	0.016
S_2_	91,838				0.008	0.008	0.009	0.009
S_3_	76,043				0.007	0.007	0.008	0.008
S_123_	221,884				0.024	0.027	0.031	0.033

## Results

### Data quality evaluation

The result of the ETL process regarding the data stored in the central repository was evaluated in terms of data quality. There were no duplicate entries found, which can be attributed to the origin relational database design as well as the lack of corresponding defects in the ETL process. There were null address values, which were intentionally not rejected during the transform stage since the field of patient address underwent anonymization (38, 39).

### Anonymity validation

The integrity of the data anonymization process was validated through the development of a simple validation tool, the object of which is to perform queries to the central repository to retrieve the anonymized data, group them by the QI attributes in order to retrieve the equivalence classes and check if there is an equivalence class with size greater than the k value chosen during the extraction stage. The application of this method proved that the data contents of the central repository do not violate the k-anonymity condition since no equivalence class consisting of fewer than k documents was found.

### Information loss evaluation

**Figure 2:**
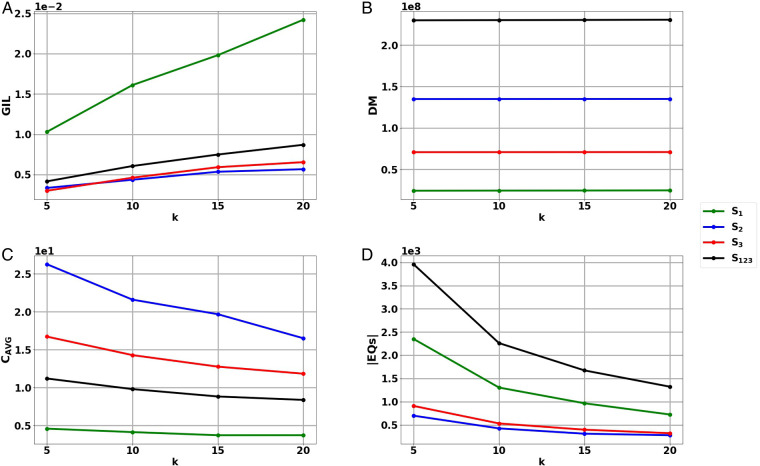
Results of the information loss metrics (**A**) Generalized Information Loss (GIL), (**B**) Discernibility Metric (DM) and (**D**) Average Equivalence Class size (C_AVG_), as well as (**D**) the number of Equivalence Classes (|EQs|) of the harmonized, anonymized data sets S_1_, S_2_, S_3_, S_123_ for the tested k values. The results are depicted in scientific format.

The generalized information loss (GIL), discernibility metric (DM) and average equivalence class size (C_AVG_) metrics (Section 2.4) were applied on the ETL output of the experimental datasets S_1_, S_2_, S_3_, S_123_ for all tested k values. The results of the evaluation can be seen in Table 2 and Figure 2.

**Table 2:** Results of the generalized information loss (GIL), discernibility metric (DM) and average equivalence class size (C_AVG_) on data sets S_1_, S_2_, S_3_, S_123_ for the chosen k values. The average values (Avg) and the standard deviation (Std) of the results have been also included.

k	GIL				DM				C_AVG_			
	S_1_	S_2_	S_3_	S_123_	S_1_	S_2_	S_3_	S_123_	S_1_	S_2_	S_3_	S_123_
5	0.0103	0.0033	0.00299	0.0042	24,269,339	134,915,704	70,783,239	229,968,282	4.5921	26.277	16.7311	11.2063
10	0.0161	0.0044	0.0046	0.0061	24,400,773	134,945,050	70,828,393	230,174,216	4.1382	21.6089	14.2938	9.8092
15	0.0198	0.0054	0.0059	0.0075	24,523,747	134,987,178	70,877,395	230,388,320	3.7269	19.6866	12.7696	8.8365
20	0.0242	0.0057	0.0065	0.0087	24,691,295	135,010,136	70,931,465	230,632,896	3.7295	16.5176	11.8447	8.3856
Avg	0.0176	0.0047	0.00501	0.0066	24,471,289	134,964,517	70,855,123	230,290,929	4.0467	21.0225	13.9098	9.5594
Std	0.0059	0.00105	0.0016	0.00195	179,732.014	42,254.338	63,785.958	285,277.319	0.41179	4.0838	2.1348	1.2483

It can be observed that GIL, DM and C_AVG_ follow the same trends as k increases regardless of the experimental dataset. More specifically, increasing k results in the increase of GIL, the increase of DM and the decrease of C_AVG_ for all tested datasets S_1_, S_2_, S_3_, S_123_.

GIL depends on the dataset QI values and the record number |T| of a given dataset (Equation 1), meaning that a smaller |T| can lead to a larger GIL value. Indeed, in Figure 2A, it can be observed that GIL takes the highest values in the smallest dataset S_1_ and lower values in the larger datasets S_2_, S_3_, S_123_. The average and standard deviation GIL values obtained for datasets S_1_, S_2_, S_3_, S_123_ were 0.0176 ± 0.0059, 0.0047 ± 0.00105, 0.00501 ± 0.0016, 0.0066 ± 0.00195, respectively.

DM depends on the number of records in each EQ, as well as the number of EQs (|EQs|) created (Equation 2). As record number |T| increases, anonymization can result in more and larger EQs increasing DM, as can be seen in Figure 2B. The average and standard deviation DM values obtained for datasets S_1_, S_2_, S_3_, S_123_ were 24,471,289 ± 179,732.014, 134,964,517 ± 42,254.338, 70,855,123 ± 63,785.958, 230,290,929 ± 285,277.319, respectively.

C_AVG_ is proportional to the record number |T| but inversely proportional to |EQs| and k (Equation 3). In Figure 2C, it can be observed that C_AVG_ takes the smallest values in dataset S_1_ with the lowest record number. The highest values occur in dataset S_2_, which is second in terms of record number and at the same time has a rather low number of equivalence classes |EQs| (Figure 2D). The fact that C_AVG_ does not take the highest values in the largest dataset, S_123_ coincides with the high |EQs| value of S_123_ (Figure 2D). The average and standard deviation DM values obtained for the datasets S_1_, S_2_, S_3_, S_123_ were 4.0467 ± 0.41179, 21.0255 ± 4.0838, 13.9098 ± 2.1348, 9.5594 ± 1.2483, respectively.

## Discussion

In this paper, an integrated architecture for the facilitation of the secondary usage of clinical data has been proposed. The MODELHealth project has aimed to enable an organization to access real health record data in a universally accepted format and carry out research at a low cost. Data was harmonized to the HL7 FHIR standard, and anonymized according to the k-anonymity principle through the Mondrian algorithm. The effect of anonymization was quantified using the generalized information loss, discernibility metric and average class size metrics. In future work and subsequent versions of the platform, extensions of k-anonymity will be considered in order to add more privacy features to the central data repository, as well as other state-of-the-art approaches, such as differential privacy.

A noteworthy challenge that was met at the stage of transformation concerned the quality of EHR data, which were characterized by high dimensionality, heterogeneity, noise and sparseness. Different codes, measure units and terminologies were often used to represent the same clinical phenotype. Therefore, the harmonization of these EHR data, initially stored in relational health center databases, to the FHIR scheme required extensive transformations through custom software.

The development of predictive models utilizing EHRs has been proposed as a promising means towards the improvement of personalized medicine and health care quality. Numerous machine learning methods have been successfully applied to patient hospitalization metadata to accomplish meaningful prediction of medical-related outcomes. Deep neural networks, in particular, have proven their ability to handle large volumes of relatively messy clinical data and have emerged as a preferred method (5, 40–44). The applicability of the MODELHealth data as input to predictive models was reassured through the development of proof-of-concept machine learning models that utilized the transformed clinical data.

## Conclusions

The secondary research use of EHR data without compromising the patients' rights to privacy is one of the most discussed topics in Health IT nowadays as well as a source of great controversy on whichever level (academic, technical, administrative, political) this discussion takes place. The results of this study add experimental data in favor of the side of the argument that adequate anonymization while preserving actionable and meaningful information can be performed on health datasets *via* proper utilization of network and data flow architectures and algorithmic tools already available in the respective literature.

## Data Availability

The project datasets and code cannot be made publicly available, because the submitted paper is part of the MODELHealth project, which has been co-funded by the European Regional Development Fund of the European Union and Greek national funds.
