# Supplementary material for: Measuring the impact of anonymization on real-world consolidated health datasets engineered for secondary research use: Experiments in the context of MODELHealth project
Source: Front Digit Health. 2022 Sep 1;4:841853. doi: 10.3389/fdgth.2022.841853 (PMC9474677; doi:10.3389/fdgth.2022.841853)
Supplement: Supplementary file 1 [file Data_Sheet_1_v1.zip › Supplementary Material/Supplementary Figure 1/Supplementary Figure 1 caption.docx]

Supplementary Figure 1. The Entity-Relationship (ER) diagram of the hospital database tables from which data were obtained during the ETL extraction stage. Some of the table columns have been excluded since they were not taken into account during the conversion of the relational objects to the FHIR standard. The table CARE_PERSON contains patient related data, CARE_ENCOUNTER, CARE_ENCOUNTER_LOCATION and CARE_ENCOUNTER_NOTES contain data related to each hospitalization, while LIS_ORDERS and LIS_TESTS refer to diagnostic tests and their results.
