# Supplementary material for: Measuring the impact of anonymization on real-world consolidated health datasets engineered for secondary research use: Experiments in the context of MODELHealth project
Source: Front Digit Health. 2022 Sep 1;4:841853. doi: 10.3389/fdgth.2022.841853 (PMC9474677; doi:10.3389/fdgth.2022.841853)
Supplement: Supplementary file 1 [file Data_Sheet_1_v1.zip › Supplementary Material/Supplementary Figure 2/Supplementary Figure 2 caption.docx]

Supplementary Figure 2. The ER diagram of the FHIR ontologies to which the EHR data was converted during the harmonization stage of the ETL process. The Foreign Keys (FK) symbolize the FHIR reference fields.
