# Supplementary material for: Measuring the impact of anonymization on real-world consolidated health datasets engineered for secondary research use: Experiments in the context of MODELHealth project
Source: Front Digit Health. 2022 Sep 1;4:841853. doi: 10.3389/fdgth.2022.841853 (PMC9474677; doi:10.3389/fdgth.2022.841853)
Supplement: Supplementary file 1 [file Data_Sheet_1_v1.zip › Supplementary Material/Supplementary Figure 3/Supplementary Figure 3.docx]

Supplementary Figure 3. A sample EHR record regarding a male patient before and after anonymization. (A) The EHR record that has been harmonized to the FHIR standard, without anonymization. (B) The sample record after harmonization and anonymization using the Mondrian algorithm. The QI attributes are the FHIR fields “address”, “birthDate”, as well as the added fields “ord_latitude” and “ord_longitute”.
