# Supplementary figures and images for: Measuring the impact of anonymization on real-world consolidated health datasets engineered for secondary research use: Experiments in the context of MODELHealth project
Source: Front Digit Health. 2022 Sep 1;4:841853. doi: 10.3389/fdgth.2022.841853 (PMC9474677; doi:10.3389/fdgth.2022.841853)

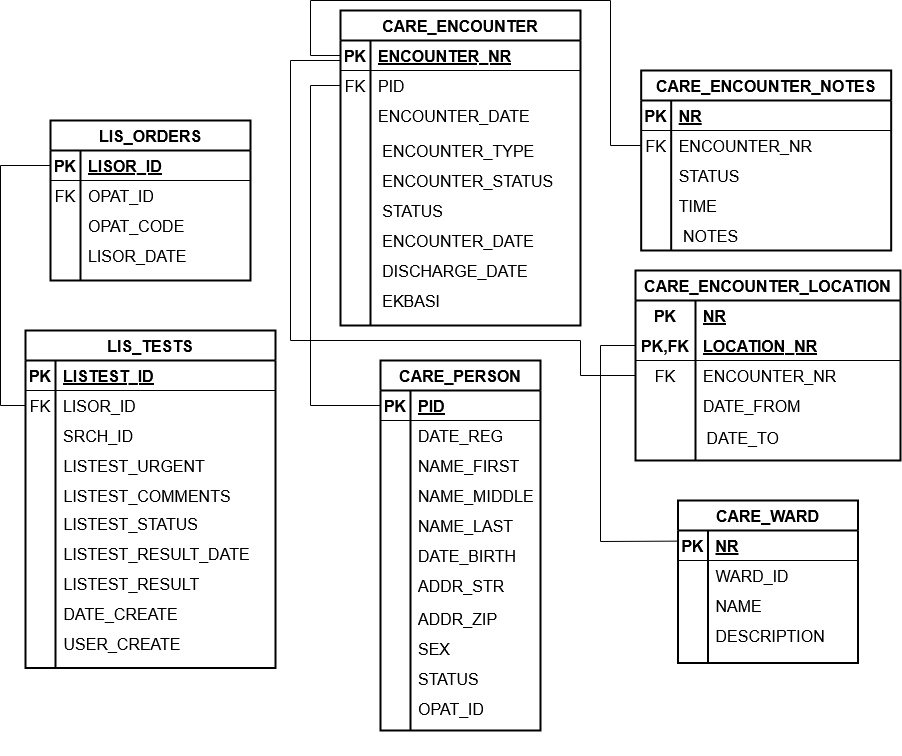

Supplement: Supplementary file 1 [file Data_Sheet_1_v1.zip › Supplementary Material/Supplementary Figure 1/Supplementary_Figure1_600dpi.png]

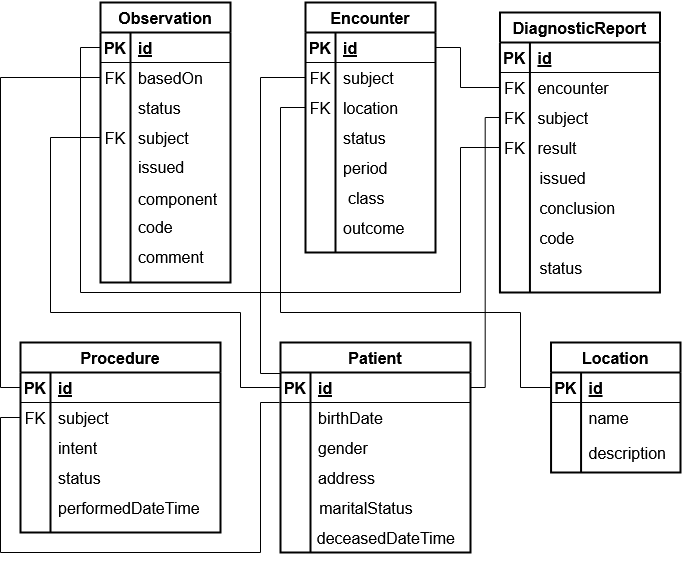

Supplement: Supplementary file 1 [file Data_Sheet_1_v1.zip › Supplementary Material/Supplementary Figure 2/Supplementary_Figure2_600dpi.png]

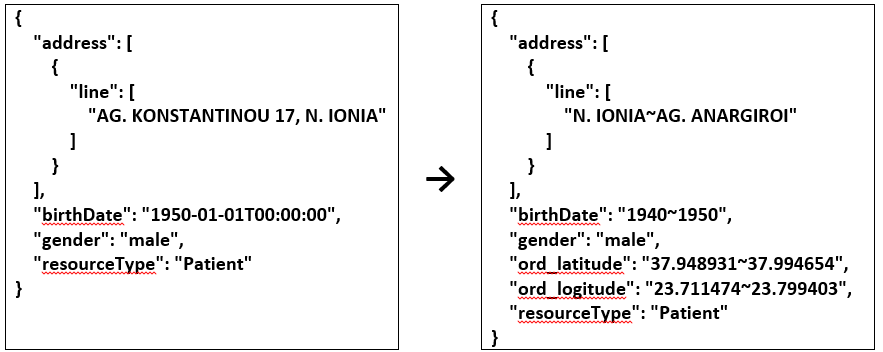

Supplement: Supplementary file 1 [file Data_Sheet_1_v1.zip › Supplementary Material/Supplementary Figure 3/Supplementary_Figure3_600dpi.png]
